# A Rare Case of a Bilateral Olecranon Fracture in a Young Adult With a Two-Year Follow-Up

**DOI:** 10.7759/cureus.66140

**Published:** 2024-08-04

**Authors:** Abhishek Nair, Mukesh O Phalak, Ajinkya K Chaudhari

**Affiliations:** 1 Department of Orthopaedics, Dr. D. Y. Patil Medical College, Hospital & Research Centre, Dr. D. Y. Patil Vidyapeeth (Deemed to be University), Pune, IND

**Keywords:** mayo classification, upper extremity trauma, complex trauma, open reduction internal fixation, anatomical plating, tension band wiring, bilateral olecranon fractures, olecranon fractures

## Abstract

Olecranon fractures are common in orthopedic wards and can be traumatic or pathological in origin. There are very few cases of bilateral olecranon fractures without any associated injuries to the long bones in the literature. We present a unique case of a young 21-year-old male who has an isolated bilateral olecranon fracture following a road traffic accident. The patient had a closed fracture of the ulna on both sides without any associated injuries or neurovascular compromise. Since the patient was young and had good muscle strength preoperatively, we planned fixation of both sides. The patient underwent open reduction and internal fixation with tension band wiring on the right side, which was his dominant side. The left side was operated on by open reduction and internal fixation with an anatomical plate. The patient was started on elbow range of motion on the right side from the second^ ^postoperative day and started basic activities such as having food independently by the 10th day postoperatively. The physiotherapy was continued in a stepwise manner, and by the sixth week, the patient had a full range of motion on both sides. The patient had resumed his activities of daily living independently by the sixth^ ^week following the surgery. Such cases are rare, and a case-based management plan must be devised for each patient, considering contributing factors such as age, bone quality, osteoporosis, underlying medical comorbidities, functional demands, and muscle strength. We demonstrated a good clinical and radiological outcome by using tension band wiring on the dominant side with a stable olecranon fracture and plating done on the non-dominant side, which had an unstable displaced olecranon fracture.

## Introduction

Olecranon fractures are common injuries in trauma wards of hospitals. They represent approximately 10% of all the fractures around the elbow in adults and present as isolated injuries most of the time [1]. They may be due to falls on stairs, road traffic accidents, falls while playing sports, etc. Olecranon fractures result from direct impact on the dorsal side of the elbow or indirect trauma because of forces generated by the strong triceps, causing avulsion fractures [2]. However, cases of bilateral olecranon fractures are rare and, most of the time, pathological in nature in association with systemic pathologies such as rheumatoid arthritis, sarcoidosis, and osteogenesis imperfecta [1,3,4].

These fractures are classified as per the universally accepted Mayo classification of olecranon fractures [5]. As per Mayo classification, type I is undisplaced olecranon fracture, type II is simple displaced olecranon fracture, which is further divided into type IIA displaced non-communited fractures, which are generally managed by tension band wiring, and type IIB displaced comminuted fracture, in which plating is the preferred line of management. Lastly, Mayo type III fractures are unstable displaced comminuted fractures of the olecranon. After careful study of the available literature, the number of reported bilateral olecranon fractures without associated injuries is sparse, making this case unique. We present a case of a young man with isolated bilateral olecranon fractures because of a road traffic accident without any significant medical comorbidities.

## Case presentation

**Figure 1:**
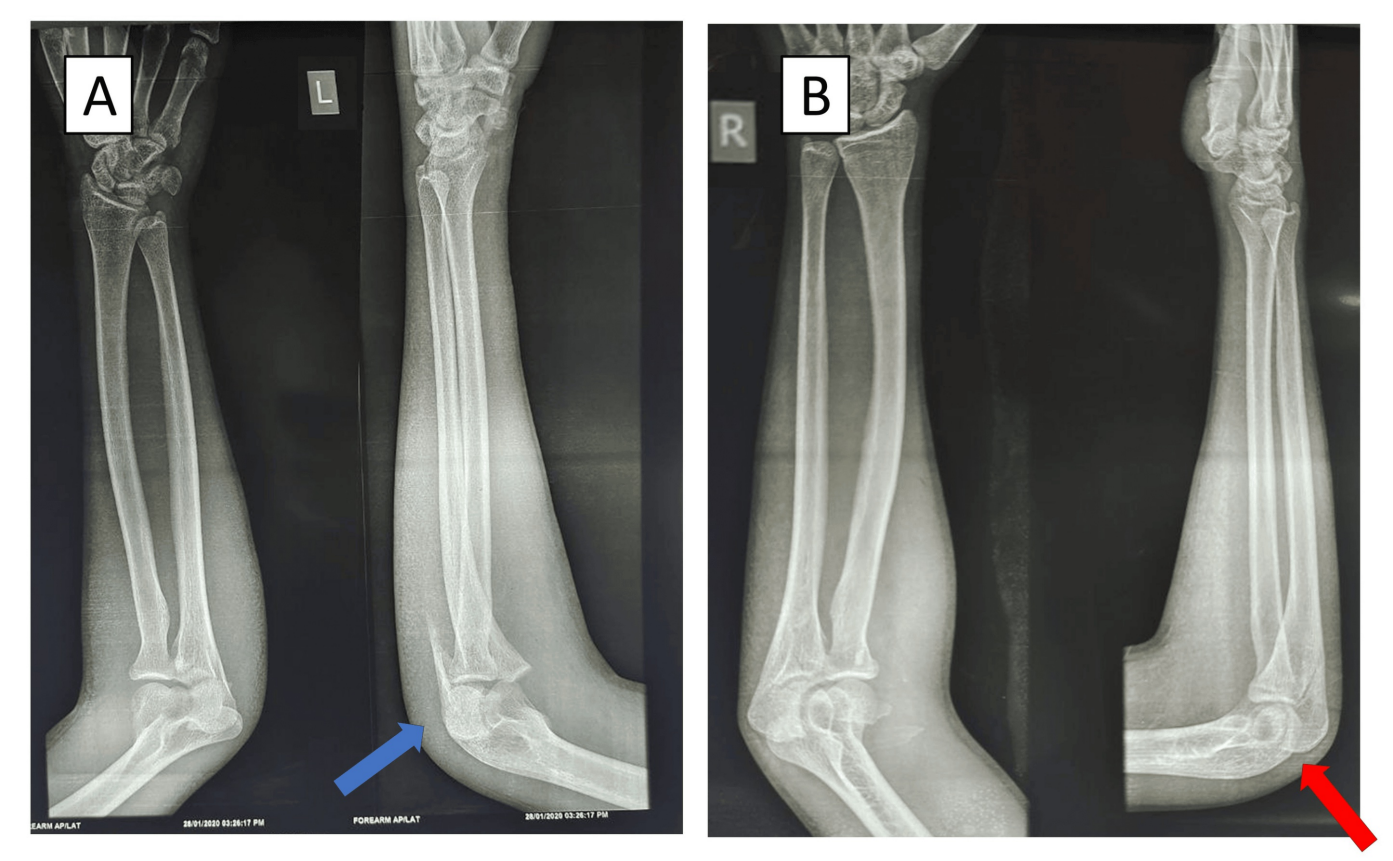
Preoperative radiographs of the left and right elbows The blue arrow shows a Mayo type II olecranon fracture on the left side, and the red arrow shows a Mayo type I olecranon fracture on the right side.

We present a case of a 21-year-old male who came to the emergency department with bilateral elbow pain following a bicycle accident during the monsoon season (road traffic accident). On clinical examination, there was severe swelling in both the elbows, and the attitude of the patient's elbows was flexion of 30°. He was unable to completely flex or extend either of the elbow joints due to pain. There were no signs of neurovascular damage. The patient was examined with anteroposterior and lateral radiographs, which revealed a bilateral olecranon fracture. The patient was given an above-elbow slab preoperatively in 30° flexion, which was the most comfortable position for the patient. Mayo's classification identified a type I fracture on the right side and a type II fracture on the left side (Figure 1).

Due to the patient's dominant right side, it was decided to do open reduction and internal fixation with tension band wire on the right side and open reduction and internal fixation with an anatomical plate on the left side in order to facilitate early mobilization. After a complete pre-anesthetic checkup, the patient was operated on the second day after the injury under general anesthesia. The main concern in this case was the operative position. At our institute, the general position for an olecranon fracture is a lateral decubitus with a bolster in the elbow to support the flexed elbow. This specific position allows the surgeon to work on olecranon fractures more easily. However, it was not feasible in this case due to the bilateral involvement of the olecranon. The patient was kept in a supine position with the shoulders abducted to 90°, externally rotated, and the elbows flexed. The upper limbs of the patient were well supported on a side arm board. This position kept the olecranon on the superior side and the radial head on the inferior side, thus making a direct posterior approach easier for the surgeon. The same surgical team sequentially operated on both sides under a tourniquet with a 250 mmHg tourniquet pressure.

The left side was operated on first. A 15-cm-long posterior skin incision was taken, and after superficial dissection, the deep fascia was incised. The ulnar nerve was identified, and the triceps was reflected. The fracture hematoma was drained, and the edges of the fracture were cleaned of the entrapped soft tissue. Under fluoroscopic guidance, the fracture fragments were reduced and held using reduction forceps. The periosteum was elevated from the shaft for appropriate plate positioning. A six-hole anatomical plate was applied and fixed with appropriate-size screws. A thorough wash with normal saline was given, closure was done in layers, and an aseptic compression dressing was given to the patient.

**Figure 2:**
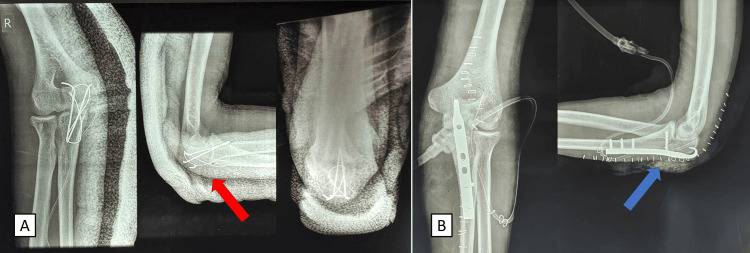
Immediate postoperative radiographs of the left and right elbows (A) Anteroposterior and lateral radiographs of the right side, with the tension band wiring done for the Mayo type I right olecranon fracture visible in the lateral view (red arrow); (B) anteroposterior and lateral radiographs of the left side, with an anatomical plate used to fix the Mayo type II left olecranon fracture visible in the lateral view (blue arrow).

Subsequently, the surgical team operated on the right side. A 13-cm-long posterior incision was taken, superficial dissection was done, the deep fascia was incised, and the ulnar nerve was identified. The fracture was identified, the hematoma was removed, and a thorough wash with normal saline was given. The fracture was reduced and fixed temporarily using pointed reduction forceps, and two 1.8 mm Kirschner wires were passed parallel to each other in a transcortical manner from the posterior to the anterior cortex. A small drill hole was made in the posterior cortex, and a pretensioned stainless-steel wire of 18 gauge was passed through it. Subsequently, this wire was passed below the intact triceps, and a figure-of-eight loop was formed. The wire was pulled to achieve compression at the fracture site, and the two ends of the wire were tied into a knot, keeping the elbow in extension, and the knot was buried laterally. The two ends of the Kirschner wire were cut and buried in the posterior side of the ulna. A thorough wash with normal saline was given, and closure was done in layers. The postoperative radiographs are shown in Figure 2.

The patient was given a plaster of Paris slab in flexion and a shoulder arm pouch bilaterally postoperatively. Adequate antibiotic coverage was administered to the patient. On the second postoperative day, as the pain subsided, passive range of motion (ROM) was started for the right side. On the fifth postoperative day, the patient had started active ROM, and by the 10th day, he was able to achieve a range of 0-45° with ease and perform basic activities such as having food with the right hand. The sutures were removed on the 14th day, and the passive ROM was started for the left side. The right side had an active range of 0-60°. On the 21st postoperative day, active ROM was started for the left elbow, and by the sixth week postoperatively, the patient had a comfortable range of 0-140° bilaterally. The patient was able to perform his activities of daily living independently without any help by the sixth week.

**Figure 3:**
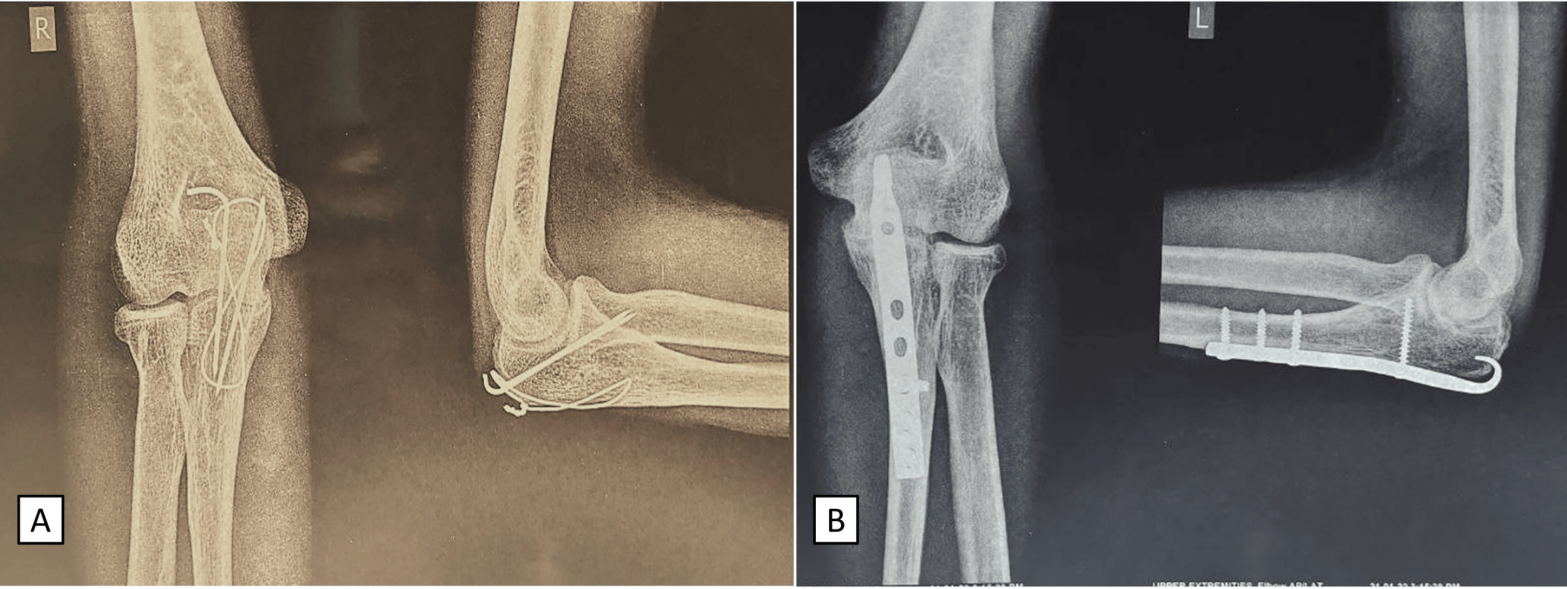
Postoperative radiographs of the left and right elbows taken six months after surgery (A) Radiograph showing the union of the right side, fixed with tension band wiring done for the Mayo type I right olecranon fracture; (B) radiograph showing the union of the left side, fixed with an anatomical plate for the Mayo type II left olecranon fracture.

**Figure 4:**
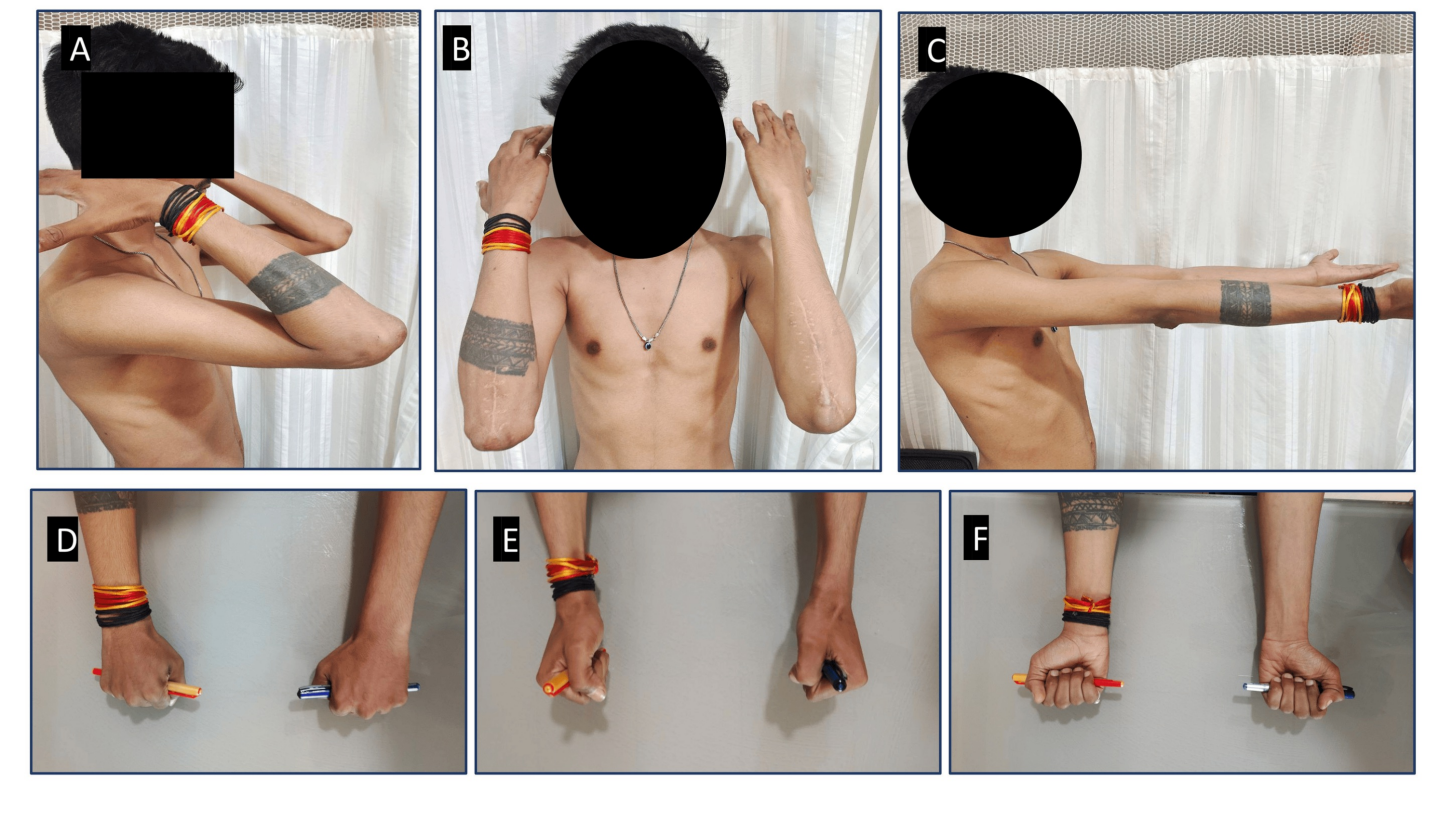
Full range of motion at six months after surgery (A) Complete flexion of the left and right elbows; (B) full flexion of the left and right elbows; (C) full extension of the left and right elbows; (D) full pronation of the left and right sides; (E) neutral position of the left and right sides; (F) full supination of the left and right sides.

**Figure 5:**
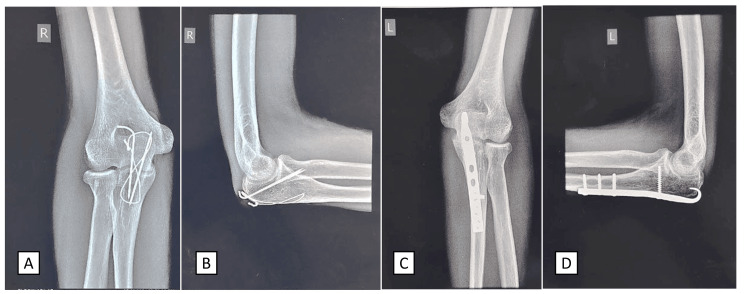
Postoperative radiographs of the left and right elbows taken two years after surgery (A) Anteroposterior X-ray view of the right elbow with tension band wiring in situ; (B) lateral X-ray view of the right elbow with tension band wiring in situ; (C) anteroposterior X-ray view of the left elbow with an anatomical plate in situ; (D) lateral X-ray view of the left elbow with an anatomical plate in situ.

Our case did not show any postoperative infection, wound dehiscence, nerve palsy, elbow stiffness, or other significant complications. The patient was followed up regularly, and at six months, there was complete union with a full ROM (Figures 3, 4). After a two-year follow-up, the patient exhibited no long-term problems and had a full ROM. Both the patient and surgeon expressed complete satisfaction with the outcome of the procedure (Figure 5).

## Discussion

Olecranon fractures are a common fracture seen in orthopedics [6]. Complicated comminuted fractures result from direct trauma, high-velocity injuries, and falls from heights, while simple transverse fractures are the result of indirect trauma [7]. Bilateral olecranon fractures can happen in road traffic accidents and high-energy injuries, but the presence of such fractures without associated fractures and injuries is extremely rare in the literature. The reported case is one such example of a road traffic accident with a bilateral olecranon fracture in a young patient without any fractures of other bones. One of the reasons for such a presentation could be direct trauma to the elbow and, secondly, excellent bone quality, which prevented other associated fracture injuries. The primary aim of managing olecranon fracture is an anatomic reduction of the articular surface, repair of the extensor mechanism, and a functional elbow joint with a full ROM [1]. There are various treatment options, such as tension band wiring, intramedullary fixation, plating, and proximal fragment excision, used to manage olecranon fractures [7].

Undisplaced fractures of the olecranon can be effectively managed conservatively by immobilizing the arm in a cast. In this case, if there was only a right-sided fracture, we could have opted for conservative management, but the bilateral involvement warranted early mobilization of at least one of the sides to reduce the dependency of the patient on others for basic activities of daily living. Tension band wiring using transcortical Kirschner wires is an excellent modality with a good outcome and is widely accepted [8]. The advantage of transcortical Kirschner wire is that it adds to the stability of the fixation by taking hold in the opposite cortex [9]. Tension band wiring thus serves as a stable fixation method with minimal instrumentation and allows early mobilization. Kirmani et al. reported a bilateral olecranon fracture in a rheumatoid patient, most probably owing to factors such as osteopenia in bones and geriatric age [3]. O’Daly et al. also reported a case of bilateral olecranon pathological fracture in a sarcoidosis patient presenting as a subacute elbow swelling bilaterally [4].

Bilateral olecranon fractures without a primary underlying cause and concomitant disease or associated fractures around the elbow or other long bones, such as the case we report, are rare in the literature. Raviraj et al. presented a young male with a bilateral olecranon comminuted fracture associated with a radial head and coronoid fracture bilaterally. Although he did not report any other associated long bone fractures, the association of a fracture of the radial head and coronoid with an olecranon fracture is significant. They managed the case through open reduction and internal fixation with compression plating on both sides. Postoperatively, the patient was given a splint for three weeks and, at 16 weeks, showed a complete ROM without any complications [10].

Kalande reported a 30-year-old carpenter who had a fall from a height of 3 m while at work. This patient was managed by tension band wiring on the left side and plating on the right side. The patient was started on active mobilization on the seventh postoperative day and, at six months, showed complete recovery [11]. We also followed a similar plan with great short-term and long-term outcomes without any complications.

The patient in our case was a young working adult with a high functional demand, good bone quality, and good healing potential; hence, he was surgically managed with open reduction and internal fixation to allow early mobilization and reduce the risk of complications to yield satisfactory clinical and radiographic results.

## Conclusions

Bilateral olecranon fractures are rare injuries. These cases are a result of high-energy trauma. They are rare, even in young patients. The management of such cases depends on the bone quality and the functional demands of the patient. Every case requires a case-based, tailored approach to choose a suitable combination from the variety of modalities of treatment to satisfy the patient's needs and provide a fully functional, painless mobile joint. In our case, we have satisfactory clinical and radiological outcomes using tension band wiring on the dominant right side and anatomical plating on the left side.
